# Diversity of Noroviruses throughout Outbreaks in Germany 2018

**DOI:** 10.3390/v12101157

**Published:** 2020-10-13

**Authors:** Sandra Niendorf, Mirko Faber, Andrea Tröger, Julian Hackler, Sonja Jacobsen

**Affiliations:** 1 Consultant Laboratory for Norovirus, Department of Infectious Diseases, Robert Koch-Institute, 13353 Berlin, Germany; NiendorfS@rki.de (S.N.); julian.hackler@charite.de (J.H.); 2 Department of Infectious Disease Epidemiology, Robert Koch-Institute, 13353 Berlin, Germany; FaberM@rki.de; 3 District Märkisch-Oderland, Health Office; 15306 Seelow, Germany; Andrea_Troeger@landkreismol.de

**Keywords:** norovirus surveillance, dual typing, molecular epidemiology, phylogenetic analysis, GII.P16 recombinants

## Abstract

Human norovirus accounts for the majority of viral gastroenteritis cases worldwide. It is a fast evolving virus generating diversity via mutation and recombination. Therefore, new variants and new recombinant strains emerge in the norovirus population. We characterized norovirus positive stool samples from one intensively studied district Märkisch-Oderland state Brandenburg with the samples from other states of Germany in order to understand the molecular epidemiological dynamics of norovirus outbreaks in Germany 2018. PCR systems, Sanger sequencing, and phylogenetic analyses were used for genotyping. Noroviruses of 250 outbreaks in Germany were genotyped, including 39 outbreaks for the district Märkisch-Oderland. Viral diversity in Märkisch-Oderland as compared to Germany was similar, but not identical. The predominant genogroup in Germany was GII with predominate genotype GII.P16-GII.4 Sydney, whereas GII.P31-GII.4 Sydney was the most frequent in Märkisch-Oderland. Genogroup I viruses were less frequently detected, regional and national. Within the sequences of GII.4 recombinants, two distinct clusters were identified with outbreaks from Märkisch-Oderland. Further analysis of sequence data and detailed epidemiological data are needed in order to understand the link between outbreaks in such clusters. Molecular surveillance should be based on samples collected nationally in order to trace comprehensive virus distribution and recombination events in virus population.

## 1. Introduction

Noroviruses were estimated to be the cause of 18% of acute gastroenteritis (AGE) cases worldwide [1] affecting people of all ages. In Germany and other industrialized countries, norovirus outbreaks are frequently associated with health- or childcare settings predominantly in the winter months from November to April.

Human noroviruses are non-enveloped viruses with positive sense single stranded RNA of approximately 7.5 kb. The genome is organized in three open reading frames (ORF). ORF1 encodes nonstructural proteins, whereas ORF2 and ORF3 encode for the major and minor viral capsid protein, respectively.

Human noroviruses are divided according to their genetic diversity into five genogroups: GI, GII, GIV, GVIII, and GIX. Recently, 18 GI and 49 GII polymerase types were proposed together with nine GI VP1 types and 29 GII VP1 variants [2]. Recombination is a common evolution strategy for noroviruses, it occurs most often as a recombination event at the junction of ORF1 and ORF2 to affect viral fitness and lead to predominance in the viral population. Point mutations within the genome particularly within the encoding region of the capsid proteins (VP1 and VP2 protein), permit the virus the escape of the host immune system [3]. The capsid consists of the VP1 protein, which contains the shell (S) domain and the protruding (P) domain. The P domain of VP1 is further subdivided into the P1 and P2 domain. The P2 region is the most variable and exposed region of the VP1 protein containing antigenic epitopes [4,5,6,7,8]. 

Since the mid-1990s, GII.4 is the most prevalent genotype worldwide and it is responsible for 70–80% of all viral AGE [9]. New GII.4 strains emerge every two to eight years [10,11]. Since 2015, a rare recombinant norovirus genotype (GII.P16) spread worldwide. In the winter months of 2016 GII.P16 was the dominant genotype in outbreaks investigated in Germany [12], detected in human stool samples as various recombination variants (e.g., GII.P16-GII.2, GII.P16-GII.4 Sydney) [12].

For better understanding of the molecular epidemiological dynamics of norovirus outbreaks in Germany, in particular outbreaks of GII.P16 variants, we chose one district in the federal state of Brandenburg in order to trace a large number of outbreaks in 2018. We were interested in the distribution of norovirus genotypes, the detection of new and rare emerging variants, and the epidemiology of the outbreaks.

## 2. Materials and Methods

### 2.1. Ethics Statement

Surveillance data and data of molecular diagnostics were collected on the basis of the German Infection Protection Act. Thus, a review by an ethics committee was not required.

### 2.2. Sample Collection

In this study, human stool samples were collected from norovirus outbreaks in the federal state of Brandenburg district Märkisch-Oderland, Germany in 2018. Stool samples were sent by local health authorities and diagnostic laboratories for genotyping purpose. In total, 66 samples were sent to the consultant laboratory for norovirus at the Robert Koch Institute (RKI) from the district for analysis.

### 2.3. PCR and Sequence Analysis

All of the stool samples were diluted in PBS (1:10) spiked with an internal extraction- and PCR-control (MS-2 phage) and the RNA was extracted from 140 µl stool suspension while using the QIAcube device. Viral RNA was extracted by QIAamp Viral RNA Mini Kit (Qiagen, Hilden, Germany) and eluted in a total volume 60 µl. 

For the first screening, RT-qPCR was conducted, as described previously [13], including improvements in primer and probe concentrations yielding a modified triplex RT-qPCR (Table 1). 10 µL mastermix containing Superscript ™ III Platinum OneStep qRT-PCR System (Invitrogen, Karlsruhe, Germany) and 2 µL extracted virus RNA was used for detection. The internal extraction control was adjusted to a crossing point (Cp) value of 28 ± 2. All of the norovirus specific primers were used in a final concentration of 416 nM; however, the NV192 + NV192a mix was adjusted to a final concentration of 208 nM. The genogroup I specific probe NV-TM9-MGB was used with a final concentration of 75 nM. The final concentration of the probe specific for genogroup II (NV-TM15-MGB) was adjusted to 150 nM. Primer and probe for internal control MS2 were published previously [14], and primers were used with a final concentration of 208 nM and MS2 specific probe of 83 nM. The RT-qPCR was performed on the LC480 system (Roche, Mannheim, Germany) with cycling conditions as follows: 15 min. 50 °C, 2 min. 95 °C, (15 s 95 °C, 32 s 60 °C), 45 cycles. Plasmid DNA (2 × 10^2^ to 2 × 10^6^ copies/reaction) was used for semi quantitative detection as well as RNA of positive controls for GI, GII and RNA for MS2 phage as a positive control for the internal control (Roche, Mannheim, Germany) [14].

In the second step the viruses were genotyped. Two RT-PCR systems were used. To obtain information of the RNA-dependent RNA polymerase (RdRp, ORF1), a previously described RT-PCR [15,16] was adapted, amplifying 331bp in the nested PCR generic for genogroup GI and GII (Table 1). The RT-PCR was performed with cycling conditions, as follows: 30 min. 50 °C, 15 min. 95 °C, (30 s 94 °C, 30 s 45 °C, 30 s 72 °C) 30 cycles, 5 min. 72 °C, 8 °C using OneStep RT-PCR KIT (Qiagen, Germany). The nested PCR was performed, as follows: 15 min. 95°C, (30 s 94 °C, 30 s 45 °C, 30 s 72°C) 30×, 5 min. 72 °C, 8 °C using HotStarTaq Master Mix Kit (Qiagen, Germany).

The second RT-PCR system (ORF2, P2 region) was adapted to previously published RT-PCR in order to analyze nucleotides of the envelope region [16] (Table 1). According to the result of the RT-qPCR the protocol for genogroup specific RT-PCRs were used. For GI a semi nested RT-PCR amplified 1116 bp. The RT-PCR was performed with cycling conditions as follows: 5 min. 55 °C, 55 min. 45 °C, 2 min. 94 °C, (15 s 94 °C, 30 s 45°C, 90 s 68°C) 40×, 5 min. 68°C, 8°C using SuperScript^®^ III One-Step RT-PCR System with Platinum^®^ Taq High Fidelity DNA Polymerase (Invitrogen, Germany). The nested PCR was performed, as follows: 15 min. 95 °C, (30 s 94 °C, 30 s 45 °C, 90 s 72 °C) 30×, 5 min. 72 °C, 8 °C using HotStarTaq Master Mix Kit (Qiagen, Germany).

For GII viruses, a RT-PCR with one PCR round was used amplifying 975bp. The RT-PCR was performed with cycling conditions as follows: 5 min. 55 °C, 55 min. 45 °C, 2 min. 94°C, (30 s 94 °C, 30 s 45 °C, 90 s 68 °C) 40×, 5 min. 72 °C, 8 °C using One-step SuperScript^®^ III One-Step RT-PCR System with Platinum^®^ Taq High Fidelity DNA Polymerase (Invitrogen, Germany).

The PCR products were analyzed with Sanger sequencing and phylogenetic analysis were performed with Geneious 11.1.5 and MEGA7 [17]. The alignment was calculated with MAFFT algorithm in Geneious. In MEGA, best fit model of substitution pattern was determined with the lowest BIC score (Baysian information criterion) and modeling of a maximum-likelihood (ML) tree was done. The reliability of the branching pattern was tested with bootstrapping (1000 replicates). Distances of evolutionary divergence were calculated with MEGA7. The P2 GII.4 tree (Figure 5) was calculated with IQ-Tree, version 1.5.3 and it was further processed with iTOL (https://itol.embl.de/). GenBank accession numbers for sequences from Märkisch-Oderland were as follows: MT584847-MT584850 for GI, MT734074-MT734105, and MT745916-MT745950 for GII sequences.

### 2.4. Surveillance Data

Symptomatic norovirus infections with laboratory confirmation have been notifiable in Germany since 2001. The detection of viral RNA by RT-PCR or detection of norovirus antigen is reported to the local public health department by the identifying laboratory. The health department completes and verifies case information according to the national surveillance case definition. Case data are anonymized and electronically transmitted to the state health department and, from there to the RKI, the national public health institute in Germany. Cases of disease without laboratory confirmation, but with an epidemiological link (occurring e.g., in outbreaks in care homes), are notifiable to the local health departments, but this case information is not passed on to the state or federal level. Thus, only laboratory confirmed cases were included in this analysis.

## 3. Results

### 3.1. Epidemiology

**Figure 1:**
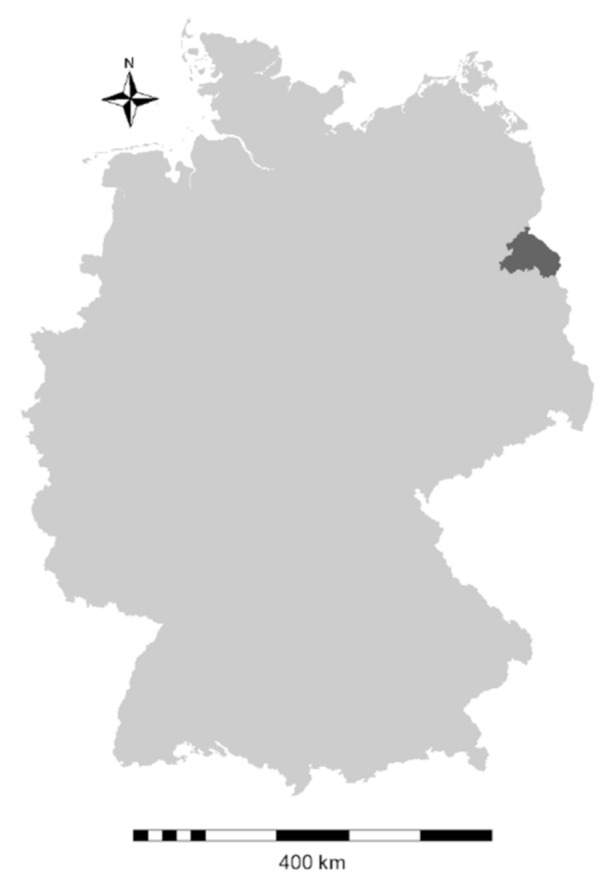
Location of the study area (district of Märkisch-Oderland, state of Brandenburg) within Germany.

In 2018, *n* = 77,583 laboratory confirmed cases of norovirus gastroenteritis were reported to the RKI, corresponding to an incidence of 93 cases per 100,000 population. In the state of Brandenburg and the district of Märkisch-Oderland, incidence was higher than the national average with 147 and 139 cases per 100,000 populations (Figure 1 shows the location of the district Märkisch-Oderland within Germany).

**Figure 2:**
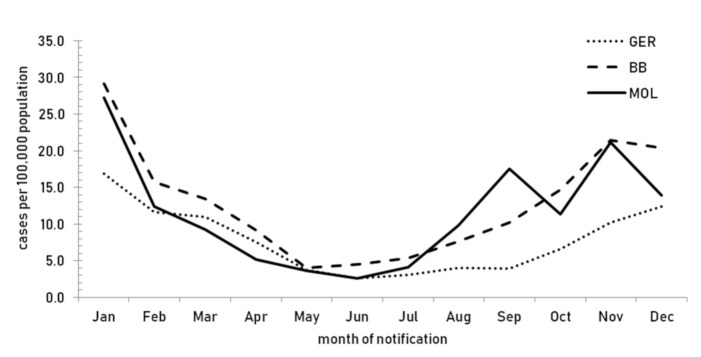
Incidence of laboratory confirmed norovirus disease by month of notification in Germany (GER), the state of Brandenburg (BB), and the district of Märkisch-Oderland (MOL), 2018.

In 2018, there was pronounced seasonality of the norovirus disease with the majority of cases occurring between September and February (Figure 2).

**Figure 3:**
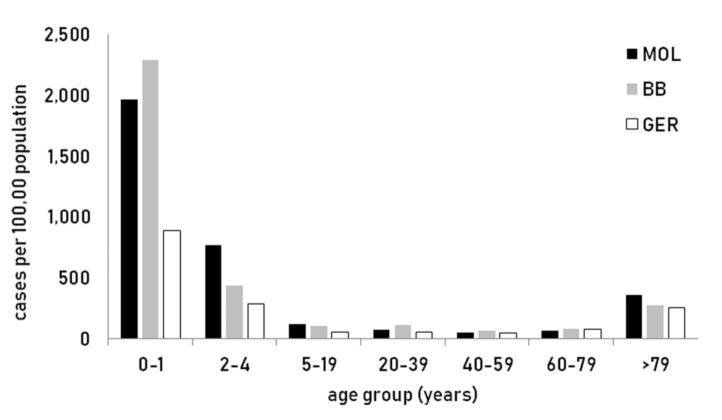
Incidence of laboratory confirmed norovirus disease by age group in Germany (GER), the state of Brandenburg (BB) and the district of Märkisch-Oderland (MOL), 2018.

Age specific incidence of laboratory confirmed norovirus disease in the district of Märkisch-Oderland was the highest in children under two years and also above the mean in the elderly population (Figure 3).

### 3.2. Distribution of Norovirus Genotypes in Germany and Brandenburg

In 2018, sequence information was available for 250 norovirus outbreaks in Germany (Table 2). Norovirus genogroup II was predominant in Germany. The predominant genotype was GII.P16–GII.4 Sydney with *n* = 72/250 genotyped outbreaks (28.8%), followed by GII.P31–GII.4 Sydney with *n =* 32/250 genotyped outbreaks (12.8%) and GII.P7–GII.6 with *n* = 27/250 genotyped outbreaks (10.8%). Next to the three frequently detected genotypes, a total of eight polymerase types of genogroup II were analyzed. Twenty percent of the outbreaks (*n* = 50/250) were caused by twelve different genotypes of genogroup I. The predominant genotypes were GI.P1-GI.1 with *n* = 8/250 genotyped outbreaks (3.2%) and GI.P2–GI.2 with *n* = 8/250 genotyped outbreaks (3.2%). Among all of the outbreaks with sequence information for both ORFs recombinant genotypes were most commonly detected (*n* = 184), 12 outbreaks were associated with recombinant viruses of genogroup I (6.5%), and in 172 (93.5%) outbreaks recombinant viruses classified as genogroup II were found.

In Brandenburg district Märkisch-Oderland, 39 outbreaks were genotyped in 2018 (39/250 outbreaks = 15.6%) (Table 2). The outbreaks were dominated by genogroup II viruses in Märkisch-Oderland (35/39 outbreaks). The predominant genotype in the district of Märkisch-Oderland was GII.P31-GII.4 Sydney with *n* = 13/39 outbreaks (33.3%), followed by GII.P16-GII.2 *n* = 10/39 outbreaks (25.6%) and GII.P21-GII.3 *n* = 6/39 outbreaks (15.4%) (Supplementary Materials: Figure S1 and S2). Whereas, GII.P16-GII.4 Sydney was most frequently observed in Germany this genotype was identified in 3/39 outbreaks (7.7%) in Märkisch-Oderland. Four of 39 outbreaks (10.3%) were associated with viruses of genogroup I, represented by genotypes GI.P2–GI.2, GIP4–GI.4, GI.P6–GI.6, and GI.P11. For the district of Märkisch-Oderland, the proportion of recombinant viruses was 87.2% (34/39 outbreaks) of all classified as genogroup II. 

### 3.3. Correlation of Norovirus GII.4 Surveillance Data and Phylogenetic Analyses in Märkisch-Oderland (Brandenburg) and Germany in 2018

**Figure 4:**
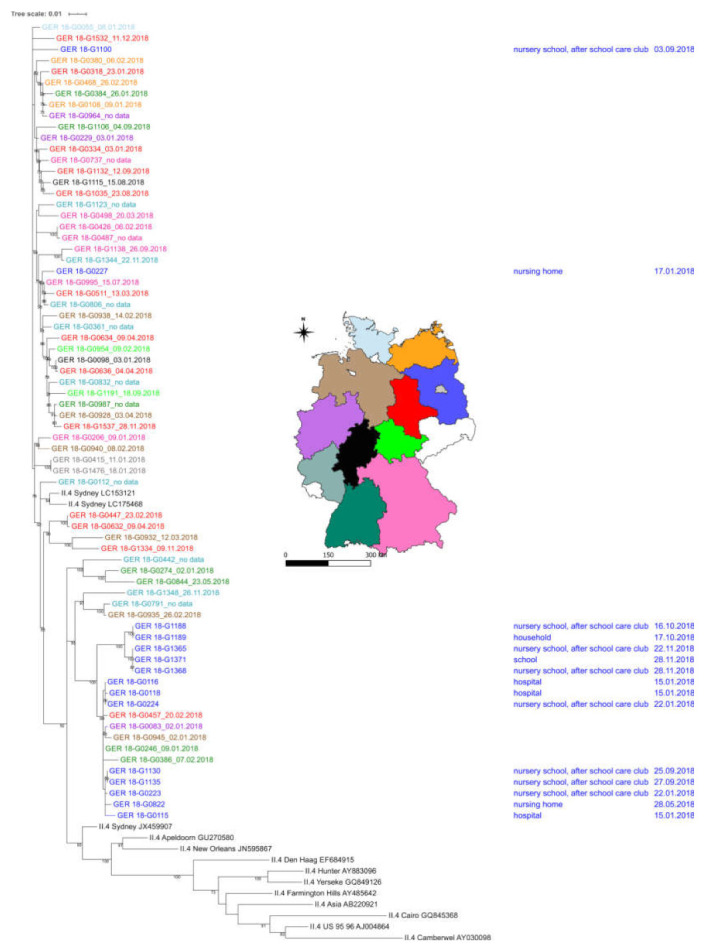
Phylogenetic tree of GII.4 sequences with nucleotide information of P2 region sampled in Märkisch-Oderland and Germany 2018 (all 83 included sequences covered a length >600bp). Blue labels in the phylogenetic tree indicate sequences from district Märkisch-Oderland, state Brandenburg, dark green labels indicate sequences from state Baden-Württemberg, brown labels indicate sequences from state Lower Saxony, red labels indicate sequences from state Saxony-Anhalt, violet labels indicate sequences from state North Rhine-Westphalia, pink labels indicate sequences from state Bavaria, grey labels indicate sequences from state Berlin, turquoise label indicate sequences from state Rhineland-Palatinate. Light blue label indicates sequences from state Schleswig Holstein, orange label indicates sequences from state Mecklenburg-Western Pomerania, black label indicates sequences from state Hesse, light green label indicates sequences from state Thuringia. Reference sequences were indicated in black: Roman numeral II.4, name and GenBank accession number. Colored states are reflected in the included map of Germany. The date next to the colored sample number indicate the date of sampling, no date means that the sampling date was unknown. On the right side of the tree the outbreak location and the sampling date in Märkisch-Oderland is given (blue label)**.**

In order to obtain detailed information about the correlation between sequence data and epidemiological information of samples collected in Märkisch-Oderland, phylogenetic analysis was performed and clustering of sequences were identified (Supplementary Materials: Figure S2). In order to determine whether clusters were limited to sequences from Märkisch-Oderland or if they were linked to other German GII.4 sequences, 83 randomized GII.4 sequences from different federal states of Germany were included in phylogenetic analysis (Figure 4). Overall, the number of nucleotide differences per sequence from averaging over all sequence pairs was 41.86 over >600 bases per sequence. The clustering of sequences in the tree was heterogenic (Figure 4). No clear clustering of sequences from same federal state was observed and no time-depended clustering of sequences was seen. Small clusters of two sequences were found from Berlin, Bavaria, Baden-Württemberg, Saxony-Anhalt. All of the analyzed GII.4 capsid sequences were linked with three polymerase types: P31, P16, and P4 2009, and the capsid sequences clustered according to the corresponding polymerase type.

Regarding sequences from Märkisch-Oderland, there were two main clusters in the phylogenetic tree. One cluster only contained sequences from Märkisch-Oderland with 4.30 base differences per sequence from averaging over all sequence pairs. In this cluster five outbreaks in a time interval from 16.10.2018 to 28.11.2018 were seen in schools, nursery school, after school care club and one affected household. In total, sixty-eight infected persons were reported in this cluster (four to 23 patients per outbreak). The polymerase type that was linked to these capsid sequences was GII.P31.

In the second cluster sequences from district Märkisch-Oderland and sequences from different federal states (Saxony-Anhalt, Baden-Württemberg, Lower Saxony, and North Rhine-Westphalia) were mixed. Outbreaks began in January (2.1.2018) and ended in September (27.9.2018), outbreaks in Märkisch-Oderland were identified from 15.1.2018 to 27.9.2018. Regarding the polymerase type of capsid sequences in this cluster, two sequences from Baden-Württemberg (GER 18-G0386, GER 18-G0246) and one sequence from Saxony-Anhalt (GER 18-G0457) represented genotype GII.P31, the polymerase type from the samples of Lower Saxony (GER 18-G0945) was not determined; all of the sequences from Märkisch-Oderland belonged to GII.P31. The base differences per sequence from averaging over all sequence pairs within this cluster were 4.38. The epidemiological data from district Märkisch-Oderland showed that 149 patients in total with six to 38 patients reported per outbreak were affected in this cluster. Two further GII.4 Sydney sequences representative for one outbreak with 57 patients and another outbreak with seven patients in Märkisch-Oderland clustered with several GII.P16-GII.4 Sydney sequences from other federal states of Germany. Both of the sequences from Märkisch-Oderland were linked with the polymerase type GII.P16. 

### 3.4. Amino Acid Changes in GII.P16 Variants

**Figure 5:**
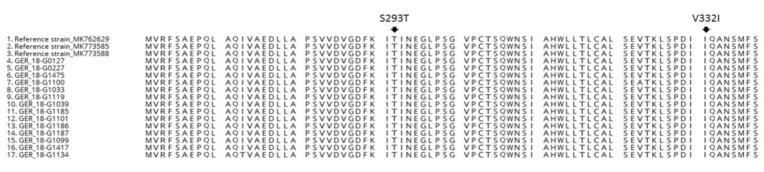
Section of the polymerase protein alignment from GII.P16 variants collected in Märkisch-Oderland with reference strains of GII.P16. Arrows indicate amino acid changes at the position S293T; V332I.

In previous studies, it was shown that the current circulating GII.P16 variants exhibit amino acid changes in the polymerase. Five mutations were found in different studies: D173E; S293T; V332I; K357Q; and, T360A [8,11,18]. In this study, fourteen RdRp-sequences (234bp) were analyzed in the ORF1 region, amino acid changes S293T and V332I were found in all sequences (Figure 5).

## 4. Discussion

It is well known that different norovirus genotypes are circulating at the same time in the same region. We traced 39 outbreaks in the district Märkisch-Oderland to study the predominant genotypes and circulation of norovirus variants in order to understand whether typing of outbreaks in one region in Germany may be representative for Germany.

In 2018, the diversity of norovirus genotypes of genogroup II was larger than the diversity of genotypes of genogroup I in Germany. The recombinant strain GII.P16–GII.4 Sydney was the most common genotype in Germany, followed by GII.P31–GII.4 Sydney. The predominant genotype in Brandenburg, district Märkisch-Oderland was GII.P31–GII.4 Sydney, followed by GII.P16–GII.2. The diversity of genotypes corresponding to GI genogroup found in Märkisch-Oderland was smaller than in Germany. Consequently, we conclude that screening for noroviruses in a small district, like Märkisch-Oderland, would not be suitable to obtain representative data for Germany although the epidemiological data (incidence, age of patients) are similar between Germany and Märkisch-Oderland. National surveillance activities are more accurate to trace new genotypes in the virus population and identity recombination events quickly.

In 2016, GII.P16 recombinant viruses started coming into focus of norovirus surveillance activity in Germany. In 2018, the proportion of all GII.P16 viruses in Germany was 40.8% with a clear predominance of GII.P16z-GII.4 Sydney (28.4%), followed by GII.P16-GII.2 (6.4%). Interestingly GII.P16 emerged in Germany in 2016 in different recombination variants: GII.P16–GII.2, GII.P16–GII.4 Sydney [12]. In 2017 the following variants were detected in Germany: GII.P16–GII.2, GII.P16–GII.4 Sydney, whereas, in 2018, five variants were circulating: GII.P16–GII.12; GII.P16–GII.2; GII.P16–GII.3; GII.P16–GII.4 Sydney (unpublished data). GII.P16–GII.4 Sydney was not only found in Germany, but also worldwide [19,20]. Ruis et al., 2017 [18] supposed that this lineage circulated in UK and USA since October 2014 and detected them in stool samples that were collected between June 2015 and April 2016.

Because GII.P16-GII.4 Sydney was the predominate genotype in Germany, it was unexpected that, in the district Märkisch-Oderland GII.P31–GII.4 Sydney was the predominant genotype, followed by GII.P16–GII.2. In the US, GII.P31–GII.4 Sydney was predominating in 2012, but it was replaced by GII.P16–GII.4 Sydney in 2015 [11]. The predominance of the genotype GII.P31–GII.4 Sydney was also reported from other countries, like China [21,22] and from Brazil [23]. It was assumed that GII.P16–GII.4 viruses would have a better viral fitness than GII.P16–GII.2 viruses [7]. For a better understanding of the norovirus diversity and predominance of GII genotypes, Parra et al. (2017) divided genotypes according to the VP1 region into different pattern of evolution: evolving strains and static viruses [24]. GII.4 viruses represented an evolving pattern with a short time span of 5.3 years between distinct variants within each genotype [24]. GII.2 viruses were described as static viruses. Following this hypothesis, the combination of the new emerged GII.P16 variants together with the fast evolving GII.4 capsid might increase the fitness of genotype GII.P16–GII.4 Sydney. This was confirmed by the predominance of this recombinant virus in Germany 2018. but not in Märkisch-Oderland. 

Analysis versus amino acid substitution were done in different studies in order to understand the successful emergence of GII.P16 recombinants. The novel GII.P16 recombinants in the US had no unique amino acid substitution in the VP1 region in comparison to the former circulating strains [11]. Nevertheless, several amino acid changes in the non-structural proteins were found: in the NS1/2 proteins, NS4 protein, and in the RdRp [11] indicating that the emergence is derived from complex interaction of mutations in different genome regions. Different amino acid changes in the RdRp of GII.P16–GII.4 Sydney viruses were observed when compared to former GII.P16 strains: D173E; S293T; V332I; K357Q; and, T360A [7,11,25]. In the present study, two mutations S293T; V332I were detected in all of the investigated GII.P16 strains. Tohma et al., 2017 [25] depicted that these mutations in the RdRp might influence the kinetics or the fidelity of the enzyme. It has been suggested that the mutation rate in combination with a high replication rate may represent the key factor in epidemiological fitness [26].

The P2 region encoding for the protruding domain of the capsid protein is the most variable region of the viral genome. This region contains the binding sites for human histo-blood group antigen (HBGA) carbohydrates and neutralizing antibodies. It is known that cross-protection among the different norovirus genotypes is limited and recurrent infections in children were seen in a study from Japan [27]. It has been shown for GII.4 viruses that amino acid changes in epitopes of the VP1 region resulted in global epidemics for decades. Antigenic drift variants of GII.4 persist for years until they are replaced by a new variant in a chronological sequential emergence [8], similar to influenza A viruses (reviewed by G.I. Parra [28]). Analysis of specific antigenic sites within the capsid protein that interact with human antibodies has given important insights into our understanding how genotype GII.4 strains escape from herd immunity and drive pandemic outbreaks. Different antigenic sites (A–G) in the P domain were identified for GII.4 variants, three of them (A, C, and G) were supposed to be drivers of selection and emergence of new GII.4 variants [8].

The phylogenetic analysis of P2 region from genotype GII.4 sequences of district Märkisch-Oderland and randomized chosen sequences from other German states revealed the clustering of sequences from Märkisch-Oderland, mostly in two groups. We only detected a number of four nucleotides differences in sequences forming clusters. Regarding all analyzed German sequences, a clear clustering of numerous sequences correlated to the region in which the samples were collected was not seen. Regarding the number of nucleotide difference in all German sequences, the difference per sequence averaging over all sequences was 30.86 (0 to 60 base differences). The question is why sequences from Märkisch-Oderland clustered so close together. Did we see different norovirus outbreaks or was there a link between these outbreak? It is known that norovirus outbreaks can be linked via asymptomatic patients. A meta-analysis of the global distribution of asymptomatic norovirus infections revealed that the prevalence rate in Europe was 18% (95% CI: 10–30%), when asymptomatic individuals were exposed under outbreak circumstances [29]. It has been discussed that GII.4 variants might originate from immunocompetent humans by inter-host transmission and the accumulation of mutations during the intra-variant periods [10]. Unfortunately, we did not have any further epidemiological information to track infection chains and find hints for asymptomatic patients. 

## 5. Conclusions

Norovirus diversity in Germany is very dynamic and it is dominated by few dominating genotypes of genogroup II; the co-circulation of additional genotypes extends this high diversity. The virus diversity of a small district, like Märkisch-Oderland, reflects the diversity in Germany to some degree, but cannot cover it completely. To assess trends of genotype distribution and facilitate the timely detection of recombination events in a country, molecular surveillance of norovirus strains should be based on samples from many parts of the country.

## Figures and Tables

**Table 1:** Modified primers and probes for detection and genotyping of norovirus genogroup I and genogroup II by RT-PCR.

Genogroup	Primer/Probe	Sequence	Localization ^a^
RT-qPCR			
I	192 (sense)	5’-GCYATGTTCCGCTGGATGC	5321–5340
	192a (sense)	5´-GCAATGTTYCGCTGGATGC	5321–5340
	193 (antisense)	5´-CGTCCTTAGACGCCATCATCA	5593–5574
	TM9-MGB probe (sense)	5´-VIC-TGGACAGGAGATCGC-MGB-NFQ	5345–5359
II	NV107e (sense)	5´-AACCAATGTTYAGMTGGATGAG	5007–5026
	NV107f (sense)	5´-AACCCATGTTCAGATGGATGAG	5007–5026
	NV107g (sense)	5′-AGGCCATGTTYAGRTGGATGAG	5007–5026
	NV107h (sense)	5′-AGCCAATGTTCAGATGGATGAG	5007–5026
	NV359 (antisense)	5′-TCGACGCCATCTTCATTCACA	5100–5080
	TM15-MGB probe (antisense)	5′-FAM-TCGATCGCCCTCCCA-MGB-NFQ	5048–5062
MS-2 phage	MS2-fwd_KL (sense)	5′-GGCTGCTCGCGGATAC	3166–3181
	MS2-rev_KL (antisense)	5′-AACTTGCGTTCTCGAGCGAT	3210–3229
	MS2 probe (sense)	5′-Cy5-ACCTCGGGTTTCCGTCTTGCTCGT-BQH2	3186–3209
ORF1 genotyping RT-PCR			
Genogroup	Primer/Probe	Sequence	localization ^a^
I and II	NV1c (sense)	5′-ATG AAC ATG AAT GAG GAT GG	4499–4518
	NV1d (sense)	5′-ATG AAT ATG AAT GAR GAT GG	4499–4518
	NV1e (sense)	5′-ATG AAT TCA ATT GAG GAT GG	4499–4518
	NV1f (sense)	5′-ATG AAT GCA ATT GAA GAT GG	4499–4518
	NV7b (antisense)	5′-GGD CCH TCA STY TTA TC	4977–4961
	NV7c (antisense)	5′-GGR CCY TCR CTY TTG TC	4977–4961
	NV7d (antisense)	5′-GGT CCT TCT GAT TTG TC	4977–4961
	NV7e (antisense)	5′-GGC CCC TCR GTT TTG TC	4977–4961
	NV7f (antisense)	5′-GGY CCT TCA GTY TTG TC	4977–4961
	NV6e (sense)	5′-ACC AYT WTG ATG CAG ACT A	4554–4572
	NV6f (sense)	5′-ACC AYT ATG ATG CTG ATT A	4554–4572
	NV6g (sense)	5′-ATC AYT ATG ATG CWG AYT A	4554–4572
	NV4d (antisense)	5′-ACY ATC TCA TCA TCA CCA	4884–4866
	NV4e (antisense)	5′-ACG ATC TCG TCR TCA CCG	4884–4866
	NV4f (antisense)	5′-ACT ATY TCA TCA TCA CCA	4884–4866
	NV4g (antisense)	5′-ACG ATC TCA TCG TCC CCA	4884–4866
ORF2 genotyping RT-PCR			
**Genogroup**	**Primer/Probe**	**Sequence**	**Localization ^a^**
GI	NV351 a (sense)	CCI CAT GTI ATT GCT GAT GT	5793–5812
	NV351 b (sense)	CCI CAC GTI ATM GCA GAT GT	5793–5812
	NV352 a (antisense)	TTC CCA CAG GCT TIA AYT G	6909–6891
	NV352 b (antisense)	TTC CCA CAG GCT TIA GYT G	6909–6891
	NV354 (sense)	ATG ATG ATG GCG TCT AAG GAC	5358–5378
GII	NV347 a (sense)	GAI GAT GTC TTC ACA GTY TCT T	5661–5682
	NV347 b (sense)	GAT GAT GTK TTC ACW GTI TCT T	5661–5682
	NV347 c (sense)	GAT GAY GTI TTC ACI GTI TCM T	5661–5682
	NV348 a (antisense)	GGT TRA CCC ARG AAT CAA A	6648–6630
	NV348 b (antisense)	GRT TMA CCC AAG AIT CAA A	6648-6630
	NV348 c (antisense)	GRT TRA CCC AIA CTT CAA A	6648-6630

^a^ Genome localization of primers and probes for norovirus genogroup I are based on the sequence of Norwalk/68/US (M87661) and for genogroup II on the sequence of Lordsdale/93/UK (X86557), for the internal control genome localization of primers and probes are based on the sequence of bacteriophage MS2 isolate MS2_ancestral (GQ153927), MGB: minor groove binder, NFQ: non fluorescent quencher, BHQ2: Black Hole Quencher 2.

**Table 2:** Frequency (number, percent) of investigated norovirus outbreaks related to genotypes in Germany 2018 and in the district of Märkisch-Oderland 2018. The predominant genotypes in Germany and in Märkisch-Oderland are marked in bold.

Detected Genotype	Number of Outbreaks in Germany 2018	% of Outbreaks in Germany	Number of Outbreaks in Märkisch-Oderland 2018	% of Outbreaks in Märkisch-Oderland
GI.P1-GI.1	8	3.2		
GI.P2-GI.2	8	3.2	1	2.6
GI.P2	2	0.8		
GI.P3-GI.3	3	1.2		
GI.P3	1	0.4		
GI.P4-GI.4	10	4.0	1	2.6
GI.P4	1	0.4		
GI.P6-GI.6	2	0.8	1	2.6
GI.P6	1	0.4		
GI.P6-GI.2	1	0.4		
GI.P7-GI.7	2	0.8		
GI.P9-GI.7	1	0.4		
GI.P11-GI.6	7	0.4		
GI.P11	1	1.6	1	2.6
GI.P13-GI.3	1	1.2		
GI.P13	1	0.8		
GII.P4-GII.4 Sydney	2	0.8		
GII.P4 2009-GII.4 Sydney	7	2.8		
GII.P6-GII.6	4	0.4		
GII.P7-GII.6	24	9.6	1	2.6
GII.P7-GII.7	3	2.8	1	2.6
GII.P7-GII.14	5	2.0		
GII.P7	5	2.0		
GII.P8-GII.8	2	0.4		
GII.P12-GII.3	1	0.4		
GII.P16-GII.12	3	1.2		
GII.P16-GII.2	16	6.4	10	25.6
GII.P16-GII.3	1	0.4	1	2.6
**GII.P16-GII.4 Sydney**	**72**	**28.8**	**3**	**7.7**
GII.P16	10	4.0		
GII.P17-GII.17	1	0.4		
GII.P21-GII.3	10	4.0	6	15.4
GII.P30-GII.3	1	0.4		
**GII.P31-GII.4 Sydney**	**32**	**12.8**	**13**	**33.3**
GII.P31	1	0.4		
total	250		39	

## Supplementary Materials

The following are available online at https://www.mdpi.com/1999-4915/12/10/1157/s1, Figure S1: Phylogenetic tree of GII noroviruses in the ORF1, RdRp region, 39 samples were collected in district Märkisch-Oderland 2018 (indicated by a dot); Figure S2: Phylogenetic tree of GII noroviruses in the ORF2, P2 region, sequence data were available from 32 samples collected in Märkisch-Oderland 2018. Dots in the phylogenetic tree indicate sequences from Märkisch-Oderland.
