# Supplementary material for: Diversity of Noroviruses throughout Outbreaks in Germany 2018
Source: Viruses. 2020 Oct 13;12(10):1157. doi: 10.3390/v12101157 (PMC7602084; doi:10.3390/v12101157)
Supplement: Supplementary file 1 [file viruses-12-01157-s001.pdf]

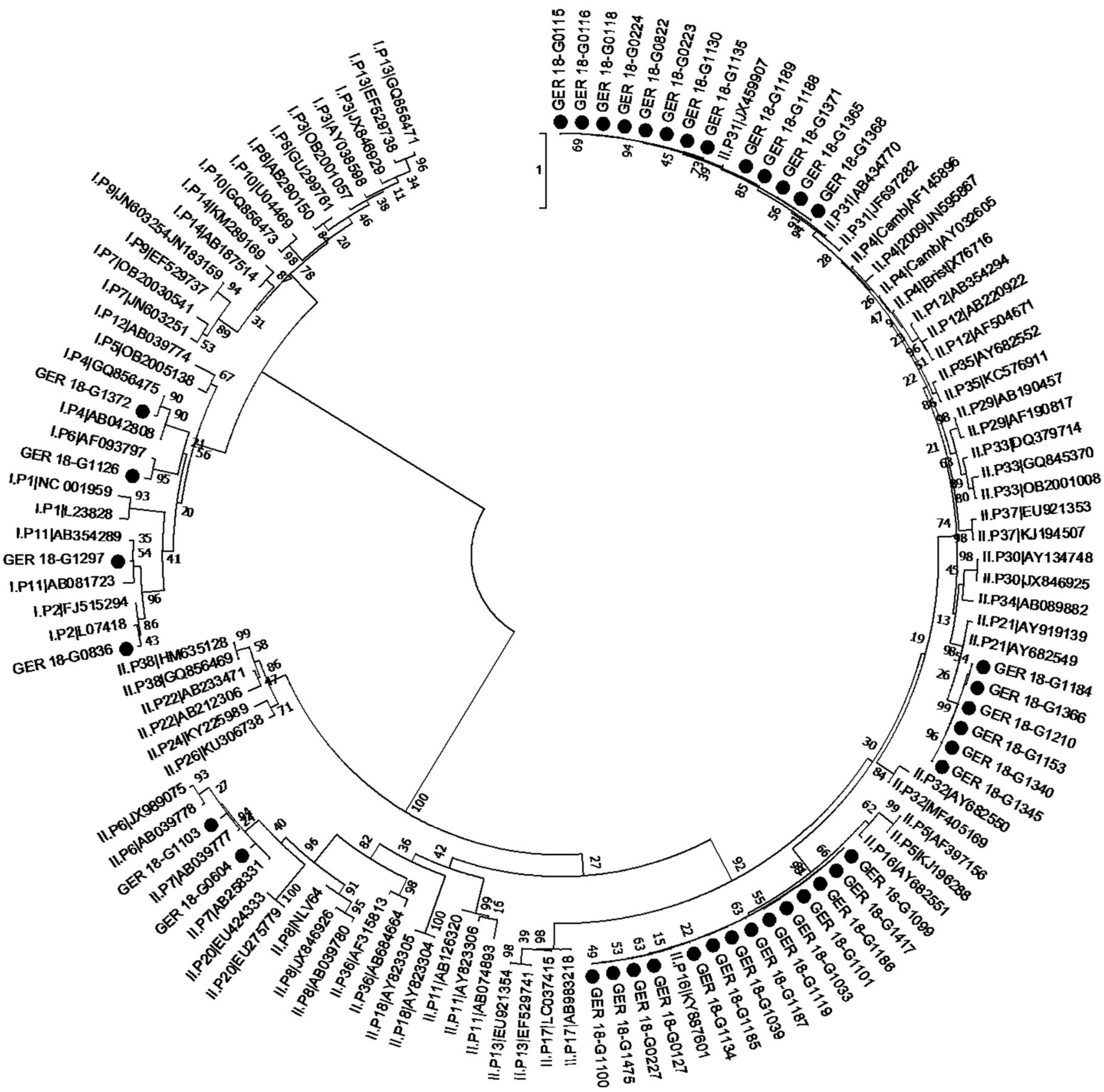

Figure S1. Phylogenetic tree of GII noroviruses in the ORF1, RdRp region, 39 samples were collected in county Märkisch-Oderland 2018 (indicated by a dot).

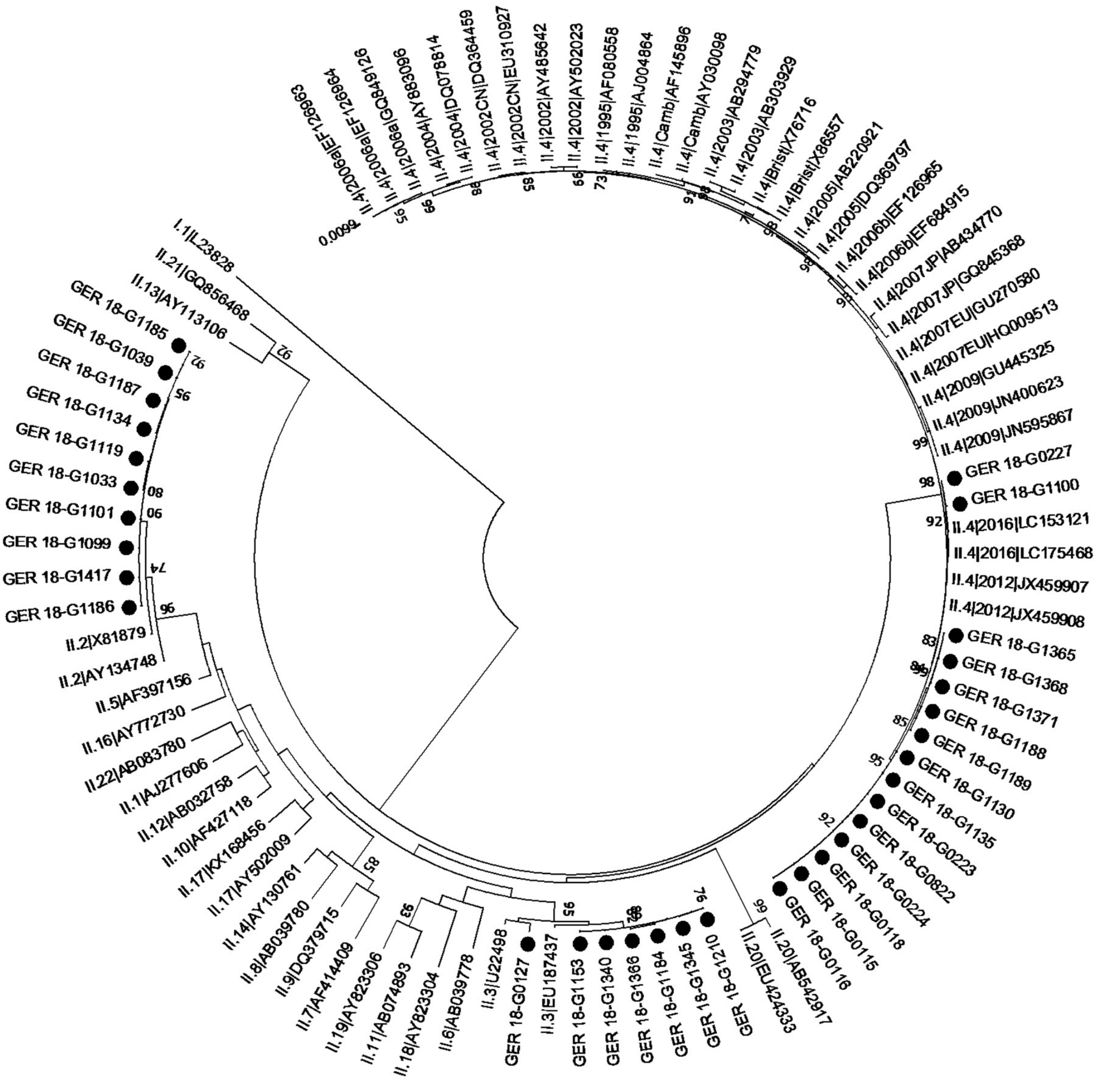

Figure S2. Phylogenetic tree of GII noroviruses in the ORF2, P2 region, sequence data were available from 32 samples collected in Märkisch-Oderland 2018. Dots in the phylogenetic tree indicate sequences from Märkisch-Oderland.
